# A Quality Improvement Approach to Increase Exercise Assessment in Survivors of Childhood Leukemia

**DOI:** 10.1097/pq9.0000000000000198

**Published:** 2019-07-29

**Authors:** Scott L. Coven, Mindy Bibart, Randall Frost, Travis Gallagher, Terri Guinipero, Amy E. Valasek, Randal Olshefski

**Affiliations:** From the * Division of Hematology and Oncology, Riley Hospital for Children at Indiana University Health, Indianapolis, Indiana; † Division of Pediatric Hematology/Oncology/BMT, Nationwide Children’s Hospital, Columbus, Ohio; ‡ Department of Quality Improvement Services, Nationwide Children’s Hospital, Columbus, Ohio; § Division of Sports Medicine, Nationwide Children’s Hospital, Columbus, Ohio; ¶ Department of Pediatrics, The Ohio State University College of Medicine, Columbus, Ohio.

## Abstract

**Introduction:**

Survivors of childhood cancer are at increased risk of treatment-related cardiovascular disease, the severity of which is impacted by the level of regular exercise. Exercise assessments (EAs) are not a routine component of follow-up care.

**Methods:**

We incorporated a quantitative EA tool into the clinic triage during follow-up visits for survivors of acute lymphoblastic leukemia. The nursing staff was surveyed on the use of the EA tool to gauge understanding and level of comfort with addressing patient questions.

**Results:**

Over 27 months, the percentage of off-therapy acute lymphoblastic leukemia patients with documented EA increased from 0% to 80%. We noted degradation in EA completions in the last 6 months of the project, which we attributed to project nursing staff transition and failure to maintain education. Interventions that improved the percentage of completed EA included the incorporation the assessment tool into the electronic medical record and weekly reminders of scheduled eligible patients. A nurse incentive plan did not impact project success. Survey results revealed that the nursing staff were comfortable with the EA and did not view the new process as hurting patient flow.

**Conclusion:**

Implementation of an EA tool into routine clinic follow-up was successful. We met the project goal of assessing greater than 50% of the follow-up patients. This work will serve as the foundation for the next phase of the project, which will be to provide education on the importance of exercise and earlier intervention when a sedentary lifestyle is identified.

## INTRODUCTION

Acute lymphoblastic leukemia (ALL) is the most common cancer of childhood, comprising 25% of all cancer diagnoses. The current 5-year survival rates for children with ALL younger than 15 years approaches 90% and 75% for adolescents between 15 and 19 years. Survivors require close follow-up due to their long-term risk of therapy-related effects.^1^ Between 60% and 90% of survivors who reach adulthood develop one or more chronic health conditions. Additionally, 20% to 80% of adult survivors experience a severe or life-threatening complication.^2–5^

The relapsed disease remains the number one cause of premature death among cancer survivors; however, cardiac and pulmonary toxicity significantly contribute to this premature risk.^6–11^ Survivors of childhood cancer show a decline in physical activity when compared with healthy controls.^12-15^ Evidence is beginning to emerge, showing a correlation between exercise level and reduced cardiovascular late effects^16^ and mortality^17^ in adult survivors of childhood cancer. Unfortunately, survivorship follow-up care does not routinely address physical activity and the benefits of regular exercise on long-term health status.

Exercise assessment (EA) has gained recent traction as a tool that can be monitored over time, similar to other vital signs.^18^ The purpose of our project was to implement an EA tool, previously validated in healthy children seeking sports physicals,^19^ during routine follow-up visits for survivors of ALL. Because quantitative and qualitative tracking of physical activity is not the norm in pediatric oncology clinics, successful implementation of the EA will provide a model that may be helpful to oncology programs looking to implement exercise interventions for cancer survivors.

## METHODS

### Project Setting and Target Population

We conducted this Quality Improvement (QI) project in the Hematology/Oncology ambulatory clinic at Nationwide Children’s Hospital in Columbus, Ohio. Approximately 150 new cases of pediatric cancer are diagnosed at our institution annually, including 25–30 patients with ALL. The target population included patients with ALL who completed therapy and were <5 years off therapy. Patients were eligible if they had an Oncology clinic visit during the study period from October 1, 2015 to January 31, 2018. There was no baseline data collected before this period. Before the project period, providers were encouraged to assess the patient’s level of physical activity. If a concern was raised by the patient, parent, or provider, an offer was made to refer the patient to a specialized Sports Medicine program designed for oncology survivors. A retrospective count of referrals to the Sports Medicine program in the 12 months before the start of the project revealed only 10 referrals.

Recommended follow-up visits for ALL patients off therapy are routine for an initial 4 years off therapy. The follow-up visits are scheduled as follows: monthly visits the first year, every other month the second year, every 3 months during the third year, and every 6 months during the fourth year. Subsequently, the patients are transitioned to our Survivorship Clinic after the fourth year.

Our ambulatory clinic serves both hematology and oncology patients with a daily clinic volume of between 40 and 60 patients. During the study period (27 months), our clinic served 20 897 visits, which averaged 770 visits per month. Off-therapy leukemia patients accounted for 5% of those visits, approximately 39 visits per month. Leukemia patients are scheduled to see providers that include fellows, nurse practitioners, and attending physicians. Nursing assignments are matched with several providers for the day on a rotating basis. We do not utilize a primary nursing system whereby certain nurses only see leukemia patients.

### Project Team and Planning

#### Project Team.

Our project team followed the Institute for Healthcare Improvement’s Model for Improvement.^20^ The core team included members from Pediatric Oncology, Sports Medicine, ambulatory nurse manager, Pediatric Hematology/Oncology fellowship program, and QI Services. We did not include a patient representative. Frontline nurses provided feedback on the progress of the project directly to the clinical nurse manager at scheduled staff meetings.

#### Key Driver Diagram

**Fig. 1.**
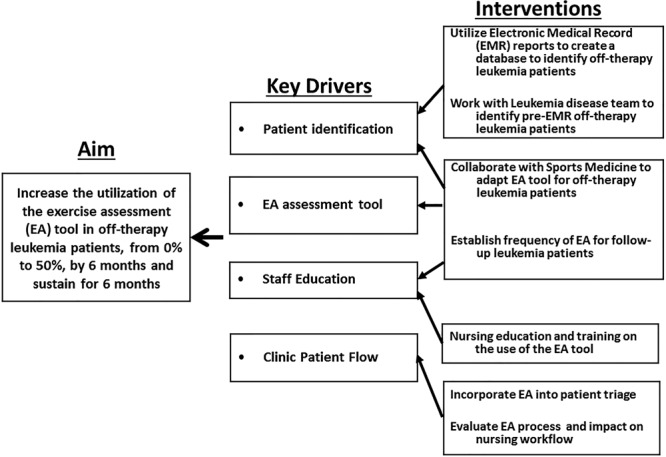
Key driver diagram depicting the project aim statement, key drivers, and interventions.

Before the start of the QI initiative, our team developed a Key Driver Diagram (Fig. 1). This QI initiative aimed to increase utilization of the EA tool in off-therapy leukemia patients, from 0% to 50%, by 6 months of implementation and sustain for 6 months. The major drivers identified by the project team included patient identification, EA tool implementation, staff education, and clinic patient flow.

### Patient Identification

At our institution, we utilize a chemotherapy ordering platform within the electronic medical record. This platform identifies patients on active treatment plans and discontinued treatment plans for any reason. The electronic query of the closed treatment protocols covered a period 7 months before the start of the project. For those patients treated before the launch of the chemotherapy ordering platform, we relied on leukemia team providers (physicians and nurse practitioners) to identify patients seen for a relapse-free follow-up. Project team members identified eligible patients before their clinic visit by cross-referencing the clinic schedule with the closed protocol list. A list of eligible patients was securely distributed to the nursing staff every week.

### Exercise Assessment Tool

**Fig. 2.**
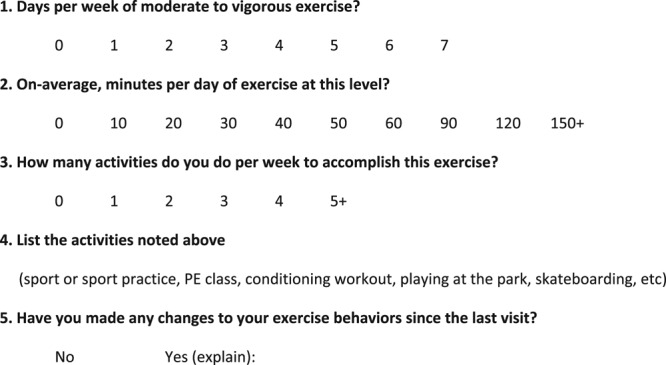
Exercise assessment questionnaire used to quantitate physical activity level in acute lymphoblastic leukemia patients who have completed therapy.

The EA tool we used to quantitate exercise activity was developed by our Sports Medicine Program^17^ (Fig. 2). In the Sports Medicine clinic, the questionnaire was designed to be completed by an athletic trainer; however, in our ambulatory clinic setting the task was completed by the frontline nursing staff. Basic demographic data, along with time off therapy, were obtained from the participants’ medical record. Survey prompts include days per week of moderate-to-vigorous exercise, minutes per day of exercise at this level, how many activities per week to accomplish this exercise, listing activities, and if there were any changes to the participants exercise behaviors since their last clinic visit. One of the important elements of the EA tool was to properly document what was considered moderate-to-vigorous physical activity. Staff were instructed to include those activities that resulted in increased respirations (panting) and/or some level of perspiration.

### Staff Education

Education on the use of the EA was provided to the nurses by a physician on the QI team during a nursing staff in-service. We used a train-the-trainer approach in that the Sports Medicine team members (A.E.V. and T.G.) reviewed the basic concepts of the EA with the oncology team member (S.C.), who in turn provided education to the frontline nurses. Reinforcement training during week 52 of the project was provided by one of the Sports Medicine team (TG), a certified athletic trainer.

### Clinic Patient Flow

During pre-project discussions with the nursing staff, concern was raised about the impact of the EA project on patient flow in a busy clinical environment. The decision was made to complete the EA during the initial nursing triage. To gauge nursing staff comfort with the EA tool, we surveyed after the initial education session.

### Project Interventions

Project process was revised utilizing Plan-Do-Study-Act (PDSA) interventions according to the Institute for Healthcare Improvement Model for Improvement.^18^ PDSA cycles during the first year of the project were intended to increase the percentage of ALL survivors with a completed EA at each follow-up visit. A project goal was to increase utilization of the EA tool in off- therapy leukemia patients from 0% to 50%. This goal was not set higher to accommodate other needs that may take priority during the clinic visit and to avoid potential survey fatigue by those families that were required to return to the clinic more frequently.

#### Interventions

PDSA 1: Weekly reminders. A team member reviewed the upcoming clinic schedule for leukemia survivors meeting eligibility for EA. By month 3 of the project, a list of upcoming patients was distributed by email to the ambulatory clinic nursing staff. At the end of each month, the number of completed EA were tallied.

PDSA 2: Incorporate EA within the electronic medical record (EMR). At the start of the project, the EA was tabulated manually on a paper form. These forms were collected by a team member weekly. During month 4 of the project, the EA questions were incorporated in the outpatient EMR navigator allowing the nursing staff to complete the EA at the same time as the required patient triage assessment.

PDSA 3: Nurse incentive. We trialed a “Mini-QI” contest where a QI indicator was chosen for 30 days. Documentation of the QI indicator was audited. Nursing staff with 100% documentation of the indicator were entered into a drawing for a gift card. The incentive plan was trialed for 6 months, starting 6 months into the QI project.

### Balance Measure

Our QI project was designed with process implementation and improvement focus. Therefore a balance measure was required to determine whether other aspects of clinic flow were negatively impacted. Nursing staff were anonymously surveyed to assess the following: (1) understanding of the EA tool, (2) whether they had adequate training, (3) whether they were able to answer all patient/parent questions specific to the exercise vital signs assessment, (4) whether the assessment could be completed in less than 5 minutes, and (5) whether they felt the assessment tool adversely affected clinic flow. The survey was sent within the first few weeks of the start of the project and after the project.

### QI Data Analysis

During the first three months of the study period, paper EA forms were collected. Once the transition to the digital format occurred, the nursing staff recorded the responses within the EMR at the beginning of the visit. This change allowed for an electronically generated monthly report to be returned to the study team for analysis.

The paper and electronic formats were tabulated and compared to the weekly distribution lists. Those patients who did not show for the clinic visit were removed from the weekly comparison. Data were analyzed utilizing a p-chart to document the overall activity of the QI project.

### Institutional Review Board

Review of the project by Nationwide Children’s Hospital Institutional Review Board was not required because the project was designated as a QI initiative. However, because of our plans to compare exercise data from ALL survivors to age-matched healthy control groups, we obtained Institutional Review Board approval.

## RESULTS

**Fig. 3.**
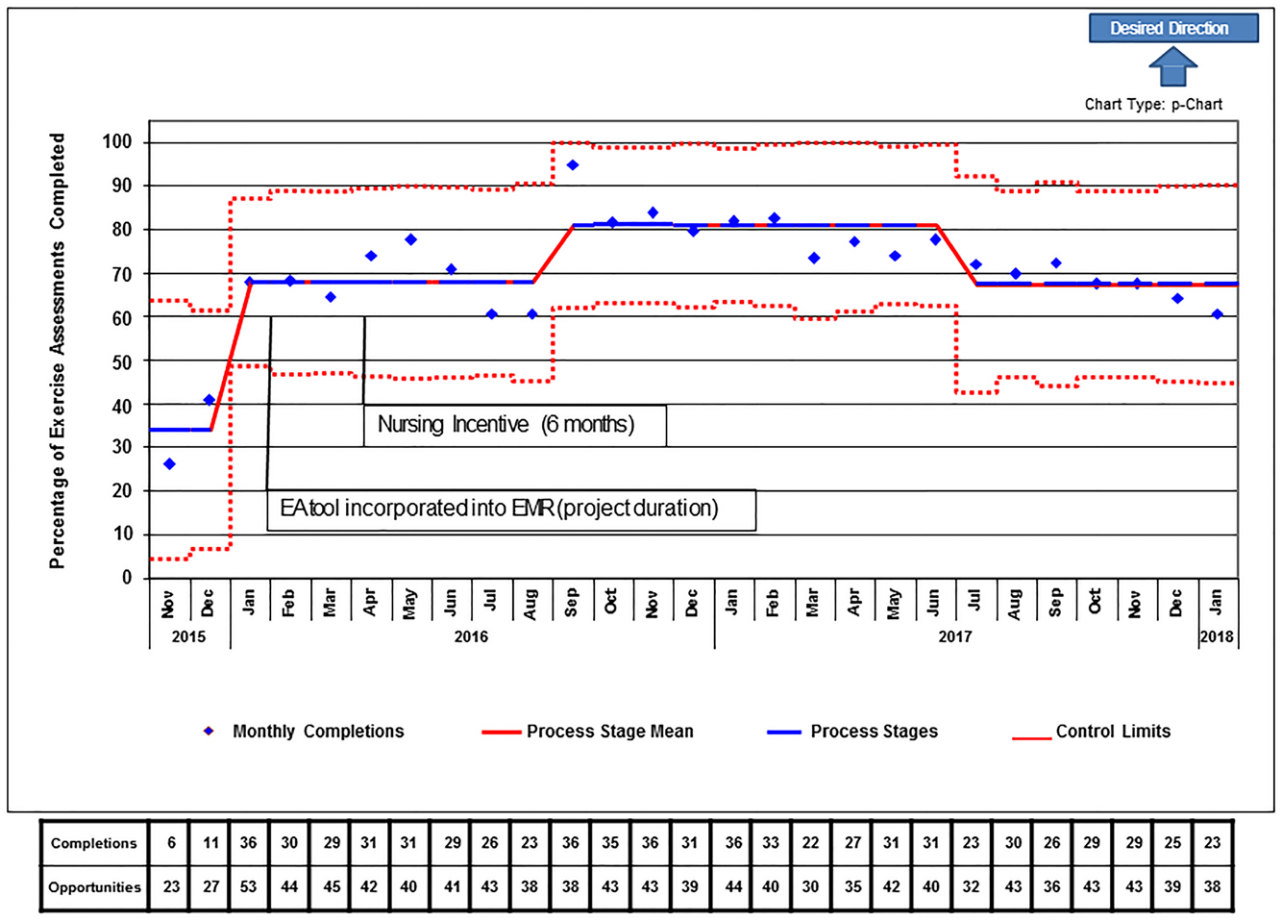
Run chart (p-chart) with time in months (*x* axis) vs percentage of eligible off-therapy acute lymphoblastic leukemia survivors completing the exercise assessment (*y* axis). Timing of QI interventions annotated in text boxes.

During the 27-month study period, we observed an increase in the percentage of completed EA above the goal of 50% (Fig. 3). The increase was sustained for the entire project period. The run chart reveals an upward shift within 3 months of the start of the project. We had two interventions during that time (PDSA 1 and 2). PDSA 1 was a successful partnership between the project team and nursing and was implemented to improve staff awareness of eligible patients before the start of the clinic day. The key intervention of transitioning the EA from paper format directly to our EMR (PDSA 2) was well received by the staff (verbal feedback from frontline nurses to the project team). The EA was easily identifiable within the “vital signs” flowsheet of the EMR. The nurses were no longer required to complete a separate paper form and could review the EA questions at the same time as the routine patient triage. The nursing staff were required to review a written update on the EMR version of the EA and participate in a brief in-service training. The intervention also improved data tabulation, which was now available through an electronic query.

The nursing incentive plan (PDSA 3) did not positively impact the EA project. After employing the incentive plan, it was determined that other competing QI and clinic operations projects were vying for the incentive dollars. Therefore, it was not feasible to sustain the incentive effort only within the EA project. In the course of the 6 months, the incentive plan was meaningfully employed in the EA project for only 2-month-long stretches.

A decrease is EA completion in the last 6 months of the project was noted. The project team concluded that the decline was due to clinic nurse transition and a failure to complete education to the new nursing staff promptly. Additionally, the project team lost a key member which coincided with a reduction in the frequency of distribution of the weekly patient lists to nursing staff. The weekly email reminders dropped off by 10% in the last 6 months of the project and ended completely in the last 1–2 months when the project team member transitioned out of our institution.

### Balance Measure: Nursing Workflow Survey

The first survey after the initial training was sent to 31 nurses, with 9 completions (29% response rate). For the final survey, the project team refined the distribution list to a smaller group of nurses with a higher likelihood of interacting with off-therapy leukemia patients. The final survey was sent to 18 nurses with 4 responses (22% response rate). The nursing staff who completed the survey stated they understood the concept of the EA, that they were able to complete the assessment in under 5 minutes, and that they were able to answer parent/patient questions (Table 1). The satisfaction held up at the end of the project as well. At the end of the project, fewer nurses completed the survey and half of those who did state they would have preferred more education on the EA tool. Because the survey was anonymous, we could not determine whether those nurses who responded with the need for more education were new hires in the clinic.

**Table 1.**
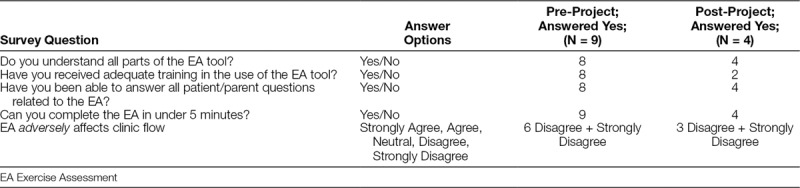
Nursing Survey Results on the Use of the Exercise Assessment Tool During Patient Triage

### Exercise Outcome Data

The primary aim of the project was to implement an EA tool during regular off-therapy follow-up visits for ALL patients. Through the 27-month project, we completed the EA during 766 clinic visits from a total of 1064 clinic visit opportunities on 132 unique patients. Survivor self-reported exercise levels were significantly less than national recommendations for childhood physical activity (Table 2). Compared with the recommended level of moderate-to-vigorous physical activity recommended by the American Academy of Pediatrics,^21^ our survivors did not meet that threshold during any off-treatment period. While survivors showed a slight increase in median exercise time during the second year in follow-up care, the level dropped off in year 3 and beyond. By year 3 of off-therapy follow-up, only 34% of the survivor visits had documented exercise levels at or above American Academy of Pediatrics guidelines.Because of the small number of unique patients and the variation in the timing of follow-up visits, occurring from monthly to every 6 months, we could not correlate improvement in self-reported exercise levels over time with the start of the EA assessments. This result is not surprising since we provided no specific intervention other than encouragement and basic education on the importance of exercise.

**Table 2.**
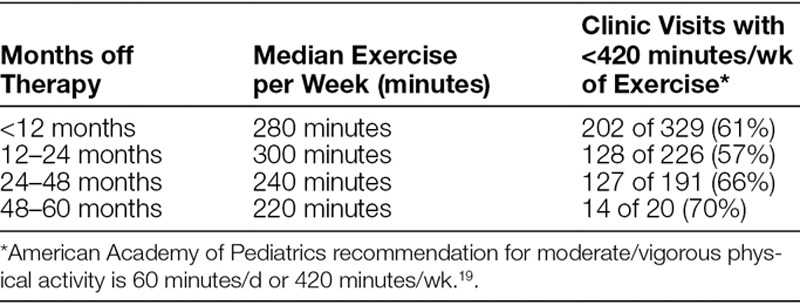
Self-Reported Exercise Levels in Survivors of Acute Lymphoblastic Leukemia

## DISCUSSION

The goal of this QI project was to consistently assess the exercise behaviors of childhood survivors of ALL. We demonstrated that physical activity assessment could be implemented and maintained in a busy clinic environment serving a mix of hematology and oncology patients. The study was originally designed to improve the baseline assessment from 0% to 50% within 6 months and sustain for an additional 6 months for a total study period of 12 months. This practice change was sustained, above goal, for an additional 17 months from the previously planned project completion.

The barriers identified after the start of the project included the use of the paper format and disseminating information to nursing staff and medical providers regarding upcoming eligible patients. We observed process improvement when we implemented a weekly preclinic reminder of eligible patients and when the paper tool was incorporated into the EMR. A staff incentive plan did not contribute to the improvement in EA completion due to the fact it could not be implemented and sustained as originally conceived.

We did not expect that we would see an improvement in physical activity following the implementation of the EA tool alone since we did not provide a specific set of interventions. The reasons associated with poor exercise activity in cancer survivors are complex.^22,23^ Therefore, understanding and overcoming the multiple barriers to physical activity will require more research.

### Limitations

Our clinic nursing structure did not allow for a consistent team to follow leukemia patients. We did an adequate job of educating the core group of nurses who triage the leukemia patients at the beginning of the project, but we did not have ongoing education through the project period. The lack of sustained training contributed to the degradation in the percentage of EA in the last 6 months of the project. Unfortunately, the project structure did not include regular huddles to address why EA were being missed or to incorporate new PDSA cycles to reverse the observed decline.

Another limitation to our project was the balance measure. We settled on front line nursing feedback via a survey. A more accurate balance measure may have included a quantitative measurement of clinic length of stay (LOS). Two barriers to tracking LOS were identified. First, the complexity of our clinic scheduling process, which is influenced by various staff and patient-related factors. Second, the potential for added strain on staff time required for tracking LOS.

### Next Steps

Our outcome data suggest that modification of physical activity behavior in a cancer survivor needs to occur before a sedentary lifestyle becomes the norm. Emerging research has demonstrated that barriers to improved physical activity will require more than verbal education and encouragement. The longitudinal documentation of exercise activity will allow for opportunities to modify physical activity behavior earlier in survivorship follow-up. Next steps for this project will require the implementation of a multi-disciplinary team approach, involving Psychology, Nutrition, Oncology, Cardiology, and Sports Medicine. The Sports Medicine program is a family-centric individualized home exercise regimen, focusing initially on the survivor’s stability and mobility. We hope to use the EA tool as part of the broader approach to developing cardiovascular, nutritional, and psychological interventions for at-risk cancer survivors.

## CONCLUSION

As employed in our project, the EA demonstrated its utility as an effective tool in measuring physical activity. The EA was quickly administered to patients and did not negatively impact clinic patient flow. We believe this process could be expanded to include other diagnoses and chronic medical conditions.

## DISCLOSURE

The authors have no financial interest to declare in relation to the content of this article.
